# Synthesis, Crystal Structure and Luminescent Property of Mg(II) Complex with *N*-Benzenesulphonyl-L-Leucine and 1,10-Phenanthroline

**DOI:** 10.3390/ma5040558

**Published:** 2012-03-23

**Authors:** Xishi Tai, Na Wei, Donghao Wang

**Affiliations:** College of Chemistry and Chemical Engineering, Weifang University, Weifang 261061, China; E-Mails: wanglihua929@163.com (N.W.); wftaixs@163.com (D.W.)

**Keywords:** *N*-benzenesulphonyl-L-leucine, Mg(II) complex, synthesis, crystal structure, luminescent property

## Abstract

A new complex [Mg(L)_2_(phen)(H_2_O)_2_](phen)(H_2_O)_2_ [L= *N*-benzenesulphonyl-L-leucine] was synthesized by the reaction of magnesium chloride hexahydrate with *N*-benzenesulphonyl-L-leucine and 1,10-phenanthroline in the CH_3_CH_2_OH/H_2_O (v:v = 5:1). It was characterized by elemental analysis, IR and X-ray single crystal diffraction analysis. The crystal of the title complex [Mg(L)_2_(phen)(H_2_O)_2_](phen)(H_2_O)_2_ belongs to triclinic, space group *P*-1 with *a* = 0.72772(15) nm, *b* = 1.4279(3) nm, *c* = 1.4418(3) nm, *α* = 63.53(3)°, *β* = 79.75(3)°, *γ* = 81.83(3)°, *V* = 1.3163(5) nm^3^, *Z* =1, *D_c_*= 1.258 μg·m^−3^, *μ* = 0.177 mm^−1^, *F*(000) = 526, and final *R*_1_ = 0.0506, *ωR*_2_ = 0.1328. The complex comprises a six-coordinated magnesium(II) center, with a N_2_O_4_ distorted octahedron coordination environment. The molecules are connected by hydrogen bonds and π-π stacking to form one dimensional chain structure. The luminescent property of the Mg(II) complex has been investigated in solid.

## 1. Introduction

The design and synthesis of metal complex materials with carboxylate ligands have attracted intense attention in recent years owing to their potential practical applications, such as molecule-based magnets, luminescence, biological properties [1,2,3]. Increasing investigations have been focused on the transition metal complex materials with carboxylate ligands [4,5,6]. Magnesium is an indispensable element in biology. It is involved in several biochemical processes and is an essential cofactor required for the activation of a variety of enzymes. Therefore, it is significant to study on the structure and characteristic coordination of magnesium carefully to understand the physiological and biochemical mechanisms of life. To the best of our knowledge, the magnesium(II) complex materials with carboxylate ligands have been much less extensively studied than other complexes. In this paper, we report the synthesis, X-ray crystal structure of [Mg(L)_2_(phen)(H_2_O)_2_]·(phen)·(H_2_O)_2_, the luminescent property of Mg(II) complex also has been investigated.

## 2. Results and Discussion

### 2.1. IR Spectra

In the infrared spectra, the *ν*_as_(COOH) and *ν*_s_(COOH) vibrations of the free ligand are at 1,659 and 1,436 cm^−1^, respectively. For the complex, the vibration observed at 1,612 cm^−1^ was assigned as *ν*_as_(COO^−^) and that at 1,398 cm^−1^ as *ν*_s_(COO^−^). It can be explained that the carboxylate oxygen atoms of *N*-benzenesulphonyl-*L*-leucine ligand take part in the coordination with magnesium atom [7]. The difference between the *ν*_as_(COO^−^) and *ν*_s_(COO^−^) band is 214 cm^−1^, indicating an unidentate carboxylate moiety. The *ν*(C = N) vibration of the free phen is at 1,589 cm^−1^, and it shifts to 1,556 cm^−1^ in the complex, indicating that the nitrogen atoms of phen take part in the coordination with magnesium atom. The bands of the -SO_2_-NH- groups at 3,248 cm^−1^, 1,320 cm^−1^ and 1,155 cm^−1^ show that there are uncoordinated atoms of the groups, because compared with the free ligand the strong absorption bands are not shifted. The new band at 421 cm^−1^ is assigned to the *ν*(Mg-O) vibration. In addition, the band at 3,423 cm^−1^ shows that the complex contains water molecules, which are accordance with the results of elemental analysis.

### 2.2. Structure Description

**Figure 1:**
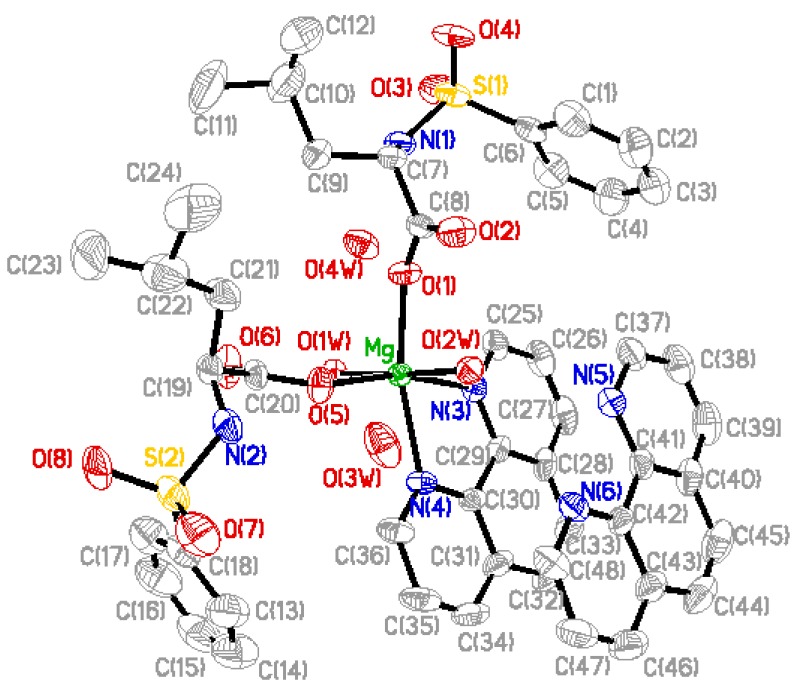
Molecular structure of the complex, where the thermal ellipsoids were drawn at 30% possibility.

**Figure 2:**
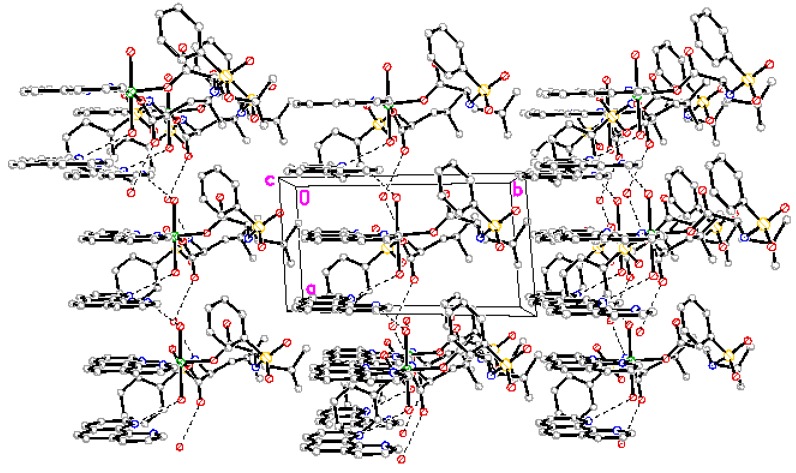
Packing of the complex.

Perspective view of the molecule and molecular packing arrangement are shown in Figure 1 and Figure 2, respectively. It can be seen that the coordination environment of the Mg(II) atom consists of two oxygen atoms from the *N*-benzenesulphonyl-L-leucine ligand, two oxygen atoms from the coordinated water molecules and two nitrogen atoms from the 1,10-phenanthroline ligand, making up a distorted octahedral environment. The coordination atoms with O(1), O(5), N(4)and N(3) atoms are situated equatorial plane and O(1w), O(2w) atoms are situated axial place. The axial bond angle O(1)-Mg-N(4) [165.95(12)°] is consistent with that of the literature structure [165.5(2)°] [8]. The distances of the Mg-O bonds are in the range of 2.020(2)~2.091(2) Å, and that of Mg-N bonds are 2.193(3) Å and 2.204(3) Å, respectively, which are similar to the Mg-O bond and Mg-N bond lengths reported previously [8]. In addition, a free 1,10-phenanthroline molecule exists in the crystal structure, and it is very important in the construction of the final structure.

**Figure 3:**
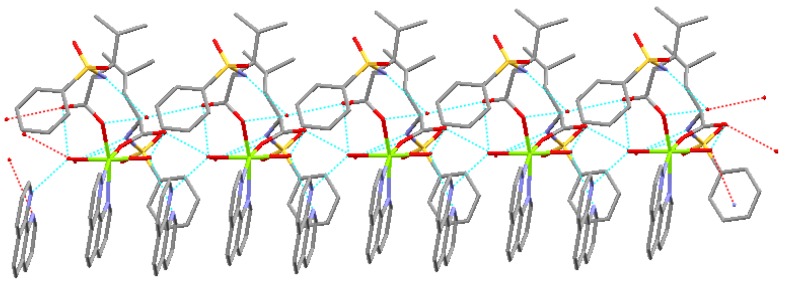
One dimensional chain structure.

The complex forms one dimensional chain structure along by intramolecule and intermolecule hydrogen bonds and π-π stacking (Figure 3).

### 2.3. Luminescent Properties

**Figure 4:**
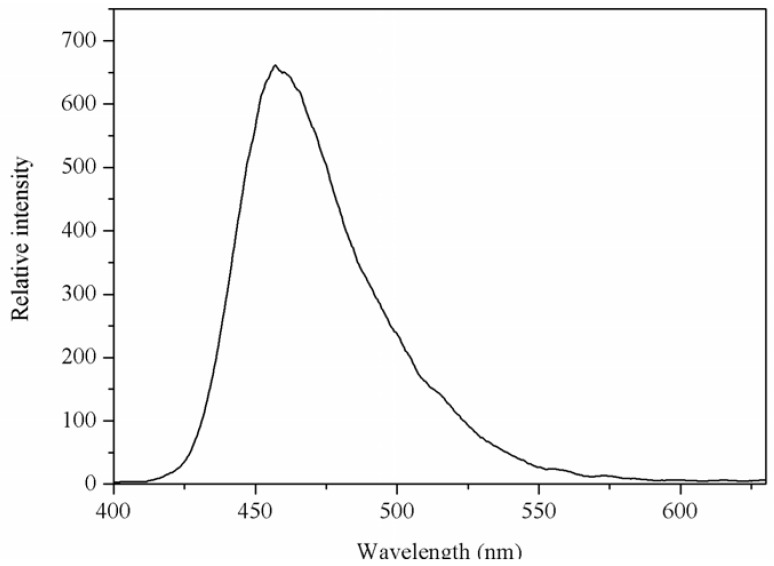
The emission spectrum of Mg(II) complex. The excitation and emission slit widths were 2.5 nm.

The luminescent spectrum of the Mg(II) complex in solid-state was measured at room temperature. The emission spectrum of the Mg(II) complex is shown in Figure 4. From Figure 4, it can be seen that the Mg(II) complex displays a luminescent emission maximum at 457 nm upon excitation at 326 nm. The emission spectrum of 1,10-phenanthroline is at 367 nm upon excitation at 267 nm [9], and the *N*-benzenesulphonyl-L-leucine ligand does not display luminescent emission maximum. Compared with the emission of 1,10-phenanthroline, the emission maximum of Mg(II) complex was red shifted. This may be the energy gap between the triplet levels of ligand and the emitting level of Mg(II) favor to the energy transfer process for Mg(II).

## 3. Experimental Section

### 3.1. Materials and Methods

The following A.R. grade chemicals were used for the preparation of the studied compound: magnesium chloride hexahydrate, benzene sulfonyl chloride, L-leucine, sodium hydroxide, 1,10-phenanthroline.

The carbon, hydrogen and nitrogen content in the newly synthesized compound were determined on a Elementar Vario III EL elemental analyzer. Infrared spectrum (4,000–400 cm^−1^) was recorded with KBr optics on a Nicolet AVATAR 360 FTIR spectrophotometer. The luminescent measurements were made on a PE LS-55 spectrometer. The crystal data was collected on a Bruker smart CCD Area Detector.

### 3.2. Synthesis of the Ligand

10 mmol (1.311 g) of L-leucine and 20 mmol (0.8 g) of sodium hydroxide were dissolved in 100 mL of water at room temperature, and added drop by drop 10 mmol (1.766 g) of benzene sulfonyl chloride by stirring at room temperature. The reaction solution was kept running for 6 h, then acidified with the solution of hydrochloric acid (V:V = 1:1) to pH = 2. The white solid precipitation were collected by filtration, washed and dried under vacuum. Yield may reach up to over 58%. Elementary analysis: calcd for C_12_H_17_NSO_4_:C, 53.14; H, 6.27; N, 5.17%; found: C, 42.98; H, 6.52; N, 5.37%. IR *ν*_max_ (cm^−1^):*ν*_as_(COOH):1659 cm^−1^, *ν*_s_(COOH):1436 cm^−1^, *ν*(N-H):3248 cm^−1^.

### 3.3. Synthesis of Mg(II) Complex

1.0 mmol (0.271 g) of *N*-benzenesulphonyl-L-leucine and 1.0 mmol (0.04 g) of sodium hydroxide were added to the 10 mL of CH_3_CH_2_OH/H_2_O (v:v = 5:1) solution. After being dissolved, 0.5 mmol (0.1015 g) of magnesium chloride hexahydrate was added to the solution. The mixture was continuously stirred for 1 h at refluxing temperature, then 0.5 mmol (0.09 g) of 1,10-phenanthroline was added to the mixture by stirring 3 h at refluxing temperature. The mixture was cooled at room temperature, and was collected by filtration. By evaporation in air at room temperature, the single crystal suitable for X-ray determination was obtained from methanol solution after 10 days. Elementary analysis: calcd for C_48_H_56_MgN_6_O_12_S_2_:C, 57.75; H, 5.61; N, 8.42%; found: C, 57.58; H, 5.99; N, 8.72%. IR *ν*_max_ (cm^−1^):*ν*_as_(COO^−^):1612 cm^−1^, *ν*_s_(COO^−^):1398 cm^−1^, *ν*(N-H):3248 cm^−1^, *ν*(H_2_O):3423 cm^−1^, *ν*(Mg-O):421 cm^−1^.

### 3.4. X-ray Crystallography

A colorless block single crystal with dimensions of 0.18 mm × 0.14 mm × 0.10 mm was placed on a glass fiber and mounted on a CCD area detector. Diffraction data were collected by *φ*~*ω* scan mode using a graphite-monochromatic Mo *Kα* radiation (*λ* = 0.71073 Å) at 293(2) K. A total of 12,805 reflections were collected in the range 3.03–27.48°, of which 9,769 were unique (*R*_int_ = 0.0472) and 7,533 were observed with *I* > *2σ(I)*. The data were corrected for *Lp* factors. The structure was solved by direct methods and refined by full-matrix least-squares techniques on *F*^2^. The structure was solved by direct methods [10] using SHELXL-97 and expanded using Fourier techniques. All non-hydrogen atoms and hydrogen atoms were refined anisotropically and isotropically, respectively. The final refinement by full-matrix least squares method was converged at *R* = 0.0506, and *wR* = 0.1328 (*w* = 1/[*δ*^2^(*Fo*^2^) + (0.0949*P*)^2^ + 0.1200*P*], *P* = (*Fo*^2^ + 2*Fc*^2^)/3, *S* = 1.052, (Δ/*σ*)_max_ = 0.000). The largest peak in the final difference Fourier map is 0.741 e/Å^3^ and the minimum peak is −0.286 e/Å^3^. Molecular graphics were drawn with the program package SHELXTL-97 crystallographic software package [11]. The most relevant crystal data for complex are quoted in Table 1, and the selected bond distances and angles are listed in Table 2.

**Table 1:** Crystallographic data for Mg(II) complex.

Crystallographic parameter	Crystallographic data
Formula	C_48_H_56_MgN_6_O_12_S_2_
Formula weight	997.42
Crystal system	Triclinic
Space group	*P−*1
*a*(Å)	7.2772(15)
*b*(Å)	14.279(3)
*c*(Å)	14.418(3)
*α*(°)	63.53(3)
*β*(°)	79.75(3)
*γ*(°)	81.83(3)
Z	1
*F*(000)	526
Temperature (K)	293(2)
*V* (Å^3^)	1,316.3(5)
Calculated density (μg·m^−3^)	1.258
Crystal size (mm^3^)	0.18 × 0.14 × 0.10
*μ* (mm^−1^)	0.177
Limiting indices	−9 ≤ h ≤ 9, −18 ≤ k≤ 16, −18 ≤ l ≤ 18
Reflections collected/unique	9769/7533
*R*_1_, *wR*_2_ [all data]	0.0710, 0.1643
*R*_1_, *wR*_2_ [*I* > 2*σ*(*I*)]	0.0506, 0.1328
Largest diff.peak and hole (e·Å^−3^)	0.741, −0.286

**Table 2:** Selected bond lengths (**Å**) and angles (°) for Mg(II) complex.

Bonds	Bond parameter	Bonds	Bond parameter
Mg-O5	2.020(2)	S1-O3	1.429(3)
Mg-O1	2.035(2)	S1-O4	1.426(3)
Mg-O1W	2.081(2)	S1-N1	1.594(3)
Mg-O2W	2.091(2)	S1-C6	1.750(5)
Mg-N3	2.193(3)	N1-C7	1.474(5)
Mg-N4	2.204(3)		
O5-Mg-O1	100.07(12)	O1W-Mg-N4	98.97(11)
O5-Mg-O1W	89.62(11)	O2W-Mg-N4	84.09(11)
O1-Mg-O1W	88.44(10)	N3-Mg-N4	75.36(11)
O2W-Mg-O5	89.76(10)	O3-S1-O4	119.1(2)
O2W-Mg-O1	88.69(10)	O3-S1-N1	106.5(2)
O1W-Mg-O2W	176.90(10)	O4-S1-N1	108.1(2)
O5-Mg-N3	165.58(12)	O3-S1-C6	107.3(2)
O1-Mg-N3	93.44(12)	O4-S1-C6	106.8(2)
O1W-Mg-N3	85.72(10)	N1-S1-C6	108.67(16)
O2W-Mg-N3	95.60(11)	O7-S2-O8	119.3(2)
O5-Mg-N4	91.95(12)	O7-S2-N2	106.5(2)
O1-Mg-N4	165.95(12)	N2-S2-O8	108.3(2)
N1-C7-C9	109.9(3)		

## 4. Conclusions

In summary, a new complex [Mg(L)_2_(phen)(H_2_O)_2_](phen)(H_2_O)_2_[L = *N*-benzenesulphonyl-L-leucine] has been synthesized and structurally characterized. The complex comprises a six-coordinated magnesium(II) center, with a N_2_O_4_ distorted octahedron coordination environment. The molecules are connected by hydrogen bonds and π-π stacking to form one dimensional chain structure. The luminescent property of the Mg(II) complex also has been investigated in solid-state.

## Supplementary Material

Crystallographic data for the structure reported in this paper has been deposited with the Cambridge Crystallographic Data Centre as supplementary publication No.CCDC 860718. Copy of the data can be obtained free of charge on application to CCDC, 12 Union Road, Cambridge CB2 1EZ, UK (E-Mail: deposit@ccdc.cam.ac.uk; Fax: +44-1223-336-033).
